# A Micro-Scale Investigation on the Behaviors of Asphalt Mixtures under Freeze-Thaw Cycles Using Entropy Theory and a Computerized Tomography Scanning Technique

**DOI:** 10.3390/e20020068

**Published:** 2018-01-23

**Authors:** Huining Xu, Hengzhen Li, Yiqiu Tan, Linbing Wang, Yue Hou

**Affiliations:** 1 School of Transportation Science and Engineering, Harbin Institute of Technology, Harbin 150090, China; 2 Department of Civil Engineering, Virginia Tech, Blacksburg, VA 24061, USA; 3 National Center for Materials Service Safety, University of Science & Technology Beijing, Beijing 100083, China

**Keywords:** asphalt mixtures, freeze-thaw damage, X-ray CT image, information entropy, microscale

## Abstract

The thermodynamic behavior of asphalt mixtures is critical to the engineers since it directly relates to the damage in asphalt mixtures. However, most of the current research of the freeze-thaw damage of asphalt mixtures is focused on the bulk body from the macroscale and lacks a fundamental understanding of the thermodynamic behaviors of asphalt mixtures from the microscale perspective. In this paper, to identify the important thermodynamic behaviors of asphalt mixtures under freeze-thaw loading cycle, the information entropy theory, an X-ray computerized tomography (CT) scanner and digital image processing technology are employed. The voids, the average size of the voids, the connected porosity, and the void number are extracted according to the scanned images. Based on the experiments and the CT scanned images, the information entropy evolution of the asphalt mixtures under different freeze-thaw cycles is calculated and the relationship between the change of information entropy and the pore structure characteristics is established. Then, the influences of different freezing and thawing conditions on the thermodynamic behaviors of asphalt mixtures are compared. The combination of information entropy theory and CT scanning technique proposed in this paper provides an innovative approach to investigate the thermodynamics behaviors of asphalt mixtures and a new way to analyze the freeze-thaw damage in asphalt mixtures.

## 1. Introduction

Asphalt mixture, which is one of the most widely used pavement materials, is a viscoelastic material that is very sensitive to the external environmental conditions [1,2]. Changes in the weather conditions, such as the alternation of freezing and thawing cycles may alter the internal structure and may consequently result in the initial damage of asphalt pavement [3,4]. Therefore, to study the distress initiation and development in asphalt pavement with freeze-thaw (F-T) actions, the determination of initiation and development of damage inside asphalt mixture needs to be firstly explored. F-T damage is one of the most serious distresses in asphalt mixtures. The difficulty associated with the identification of F-T damage in asphalt mixtures at different scales has significantly hindered the analysis on the comparison of macro-scale properties (such as strength, modulus, and macro-scale air voids content) before and after F-T cycles in asphalt pavement design. Nevertheless, consider the importance of this kind of distress, the influence of F-T action on the degradation of asphalt pavements has been analyzed by many researchers by utilizing macro-scale analysis. Xu et al. [5], Ozgan et al. [4], and Feng et al. [6] reported that after eight cycles of F-T action, the air voids in partially saturated asphalt pavement can increase by 4~12% while that in full saturated asphalt mixture can increase by 54% for the same experimental conditions. Ameri et al. [7] and Amini and Tehrani [3] adopted the resilient modulus, Marshall strength and indirect tensile strength to analyze the effect of salted water and anti-stripping additive on the performance degradation of asphalt pavement in seasonal frozen area. Wang et al. [8] compared the texture and skid resistance development to demonstrate the effect of F-T cycles on the short- and long-term development of skid resistance.

Advanced technologies, such as micro-mechanics finite element modeling technology and X-ray computerized tomography (X-ray CT) technology, allow the F-T damage in asphalt mixtures to be characterized from a micro scale perspective. Using X-ray CT images, Xu et al. [5,9] pointed out that the internal structure under F-T action mainly degraded in three ways: existing voids expansion, adjacent voids merger, and new voids formation. Especially, the F-T damage provided more flow path for the moisture penetration which would aggravate the damage. Shakiba et al. [10] simulated the thermos-hygro-mechanical response of asphalt pavement by utilizing a three-dimensional finite element microstructural model reconstructed from X-ray CT images and depicted the progressive damage process in asphalt pavement. Lamothe et al. [11] used strain gauges to detect the strain distribution on sample surface during F-T cycles. They reported that comparing to brine, samples partially saturated with water had a more significant elongation, dilation and contraction by formation and melting of ice under F-T cycles.

In conclusion, although there has been remarkable progress in this area, efforts done in previous studies in the discussion of F-T damage of asphalt mixtures are still limited in using some indirect indexes in both micro- and macro- scales without much emphasis on the direct evaluation method, which could give visual evidence for the understanding of deterioration of asphalt pavement in cold regions. To solve this problem, two types of asphalt mixtures were proposed in the presented test analysis. Specimens were scanned before and after F-T cycles to collect CT graphics for the recognition of the changes in pore structures. A micro-scale energy analysis method—information entropy method was also introduced into the analysis of F-T damage in asphalt mixtures. The information entropy was calculated from the CT graphics before and after F-T cycles and then compared with the changes in the micro-scale pore structure to verify the possible damage in the F-T cycles. The influence of test conditions on the F-T damage propagation in asphalt mixtures were also discussed on the basis of lab experimental results.

## 2. Material and Methods

### 2.1. Asphalt Mixture Preparation and Experimental Condition

**Figure 1:**
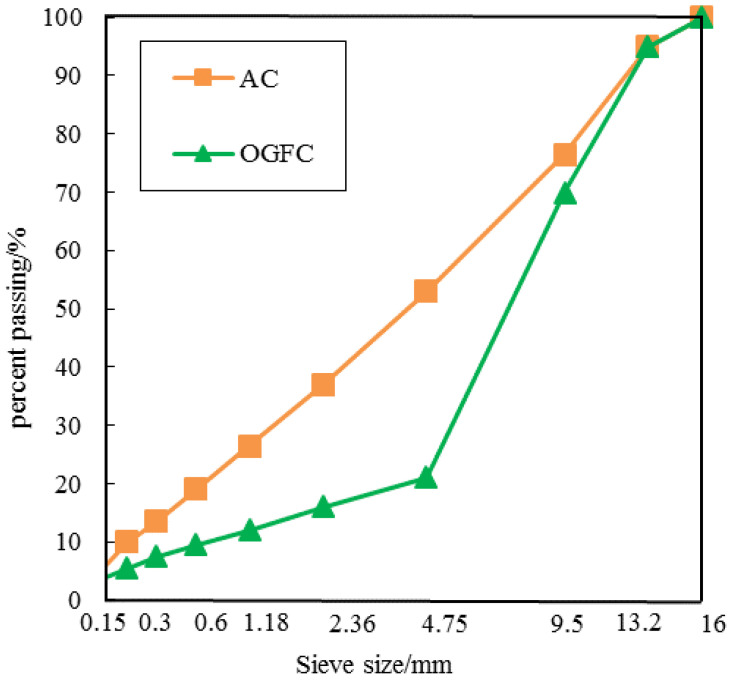
Aggregate gradations for two types of asphalt mixtures.

Two typical kinds of asphalt mixtures, a dense-graded asphalt mixture (AC) with a Superpave asphalt binder PG 58-22, and an open-graded friction course (OGFC) with 80/100 penetration grade asphalt, were selected for the experiment. The basic properties of this asphalt were shown in Table 1. The nominal maximum aggregate size for two asphalt mixtures was 13.2 mm. The basic properties of aggregate were shown in Table 2 and Table 3. The two asphalt mixtures were obtained from the Heilongjiang Department of Transportation (HDOT). A summary of gradations of the two mixtures was illustrated in Figure 1. All specimens were prepared by the Superpave gyratory compactor into cylinders with the diameter of 100 mm and the height of 63.5 mm. The design porosity of asphalt mixture were set to be 3.5% for AC mixture and 21% for OGFC mixture which were similar to actual void content used in field.

For damage analysis, in total, 16 specimens were conditioned under F-T experiments with four replicates for each condition, as illustrated in Table 4. Two F-T experiment procedures were adopted to analyze the effect of environmental condition on the F-T damage in asphalt mixtures. The control group labeled by “T-1” indicated that the samples were firstly placed in the moisture and vacuum saturated with 98 kPa residual pressure for 15 min, then put into a refrigerator to be frozen in the air for 16 h with an ambient temperature of −18 °C, and finally thawed in the moisture at 20 °C for 12 h. Unlike the samples conditioned with moisture in T-1, “T-2” referred to the sample conditioned in the humid air. In T-2 test procedure, the specimens were placed in an environment chamber with the 20 °C of air temperature and relative humidity of 90% for 15 days before the first F-T cycle to make a uniform initial humidity field. Then, the samples were placed in the air with a temperature of −18 °C for 16 h and thawed in a relative humidity of 90% for 12 h.

To understand the F-T damage from micro scale, specimens were scanned before the F-T tests to investigate the initial pore structure. Then the samples were subjected to 18 successive F-T cycles under two different conditions. After 3, 6, 9, 12, 15, and 18 F-T cycles, damaged samples were rescanned to capture the changes in pore structure by using X-ray CT.

### 2.2. X-ray Scanning and Internal Structure Extraction

The samples were scanned before and after F-T test with a compact X-ray mini-focus computed tomography (CT) system (GE, Phoenix X-ray, Hannover, Lower Saxony, Germany) for 2D X-ray inspection and 3D CT at a voltage of 180 kV, and an X-ray tube current of 100 mA and with a horizontal resolution of 0.015 mm/pixel. The asphalt mixture specimens were scanned horizontally with a slice interval of 0.064 mm and a field view of 110 mm by 110 mm. The pixel resolution of images was 72 ppi. The sample vertical axis was paralleled to the rotation axis of the scanning gantry. The captured X-ray CT images were preprocessed before three dimensional reconstruction of the microstructure of sample, including image quality improvement, noise filtering, and extraction of interest object. All the procedures were carried out by utilizing a commonly used image processing software, namely VG Studio (Volume Graphics, Hedelberg, Baden Wurttemberg, Germany). The overall void content error measured by CT is around 5%. In consideration of the moisture swell during freezing process, image slices were obtained with the numerical algorithm suggested by Hassan et al. [12] to guarantee that the slice images acquired at different times were exactly at the same position.

The pore size and morphology and connectivity of the pore space were the fundamental characteristics of internal structure of asphalt mixtures that controlled asphalt mixture properties [13,14,15]. As suggested by Masad et al., Hassan et al., and Shaheen et al. [12,16,17], a limited set of geometric descriptors could be used to depict the complex structure of asphalt mixtures. The common used geometric descriptors were: (i) void content; (ii) number of void; (iii) void diameter; and (iv) connective void content, which characterized the connectivity among pores and the branching of pores, that was, the number of pore channels.

In this analysis, the variation in the pore structure indexes of asphalt mixture samples were quantitatively discussed along the height direction by the (connective) void content, void diameter, and void number. The algorithm developed by Kutay et al. [18] for the calculation of pore structure characteristics in asphalt mixtures was employed in this work. The calculation accuracy of the above mentioned parameters has been verified by Xu et al. [5], and therefore applied in this analysis.

### 2.3. Information Entropy Analysis from X-ray CT Image

F-T damage in pavements is an energy exchange process with the external environment and results in changes to the thermodynamic entropy of asphalt mixtures. However, thermodynamic entropy is difficult to measure quantitatively and directly by using macro-scale experimental analysis. Meanwhile, information entropy, also named as Shannon entropy, is a mathematical measure about the randomness degree in one set of data. A greater randomness means the higher entropy, and greater predictability always means the lower entropy. If the thermodynamic system of asphalt mixtures was taken as a specific information system, the micro-scale distribution of molecules in the system corresponded to a possible information event, and all the micro-scale distribution of molecules constituted the macroscopic property of information. Therefore, the information entropy was essentially the same as the thermodynamic entropy.

Energy exchanges between the material and outside results in material damage. The change of energy parameters can directly reflect the energy change of materials. Material damage and energy changes have certain nonlinear characteristics, and information entropy has these characteristics [19]. Information entropy analysis has been widely used in civil engineering area. Van Siclen [20] pointed out that the information entropy function provided a sensitive measurement of the complexity of a multi-component materials system from both micro- and macro- scales. Bao et al. [21] treated cracked concrete structures as dissipative structures and investigated the relation between the propagation of cracks and the information entropy in concrete structures by using micro-scale experimental and numerical analysis. Su et al. [22] analyzed the structure reliability of concrete-faced rockfill dams (CFRD) by combining finite element method with the theory of information entropy, in which, information entropy was adopted to represent the reliability of CFRD. Regarding asphalt mixtures, Zhang et al. [23] introduced information entropy into the determination of damage degree of asphalt mortar in various moisture conditions utilizing CT images, and the feasibility of the novel method was verified by experimental results. Therefore, the information entropy is adopted in our analysis and calculated from the collected images following Equation (1):

(1) $$H=-\sum_{i=0}^{255}P_{i}\cdot \ln P_{i}$$

where *H* is the information entropy from CT images; and *P_i_* represents the proportion of pixels of *i* in the whole range of the gray level in X-ray CT images. The calculation algorithm proposed by Zhang et al. [23] was adopted. And in this research, information entropy was calculated using MATLAB (MathWorks, Natick, MA, USA).

## 3. Results and Discussion

### 3.1. Thermodynamic Behaviors of Asphalt Mixtures under F-T Cycles

#### 3.1.1. Pore Structure Degradation under F-T Cycles

**Figure 2:**
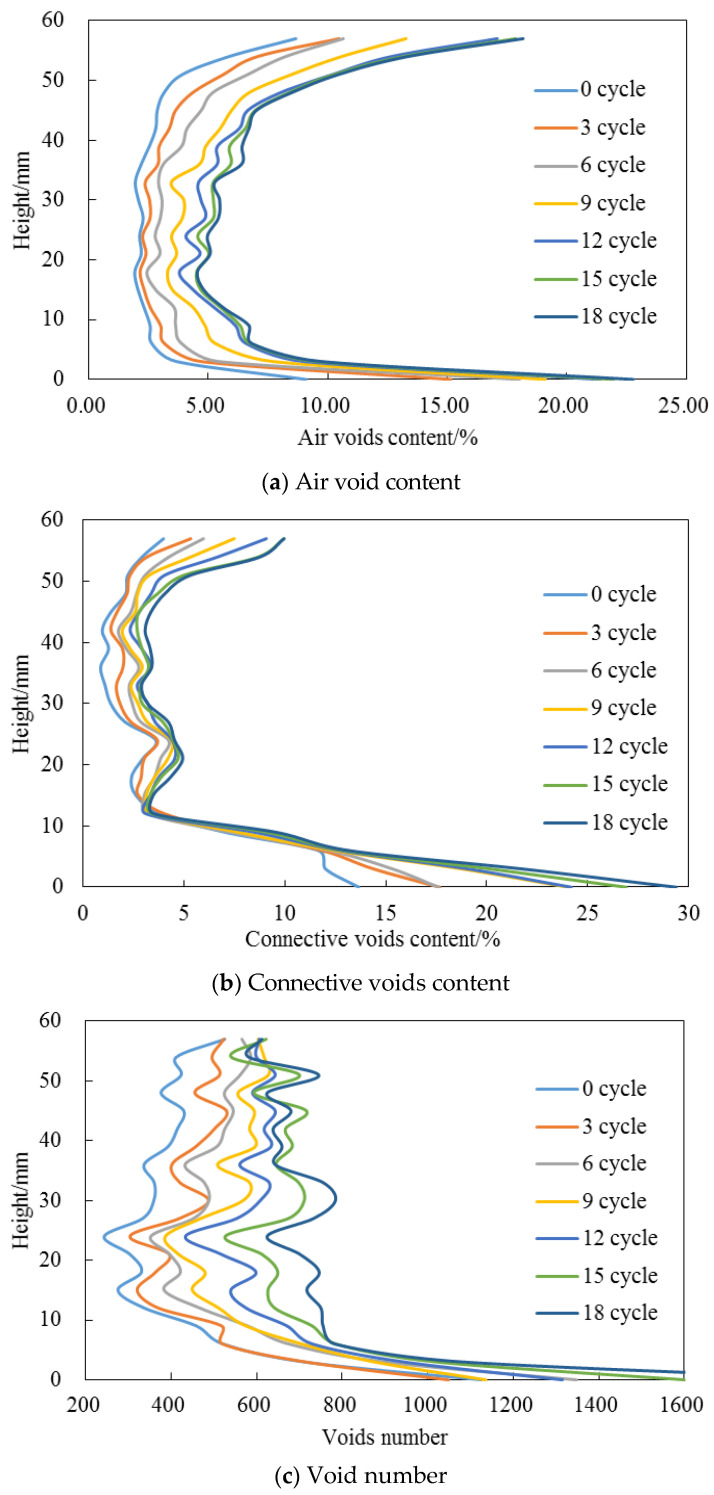

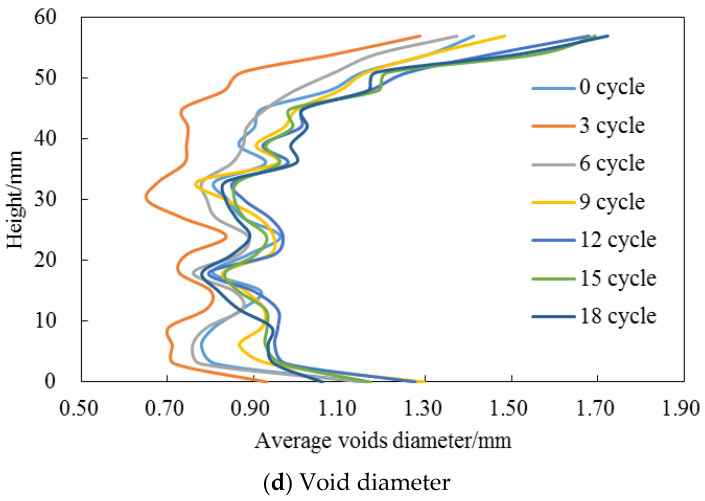
Pore structure degradation for AC mixtures under F-T cycles.

**Figure 3:**
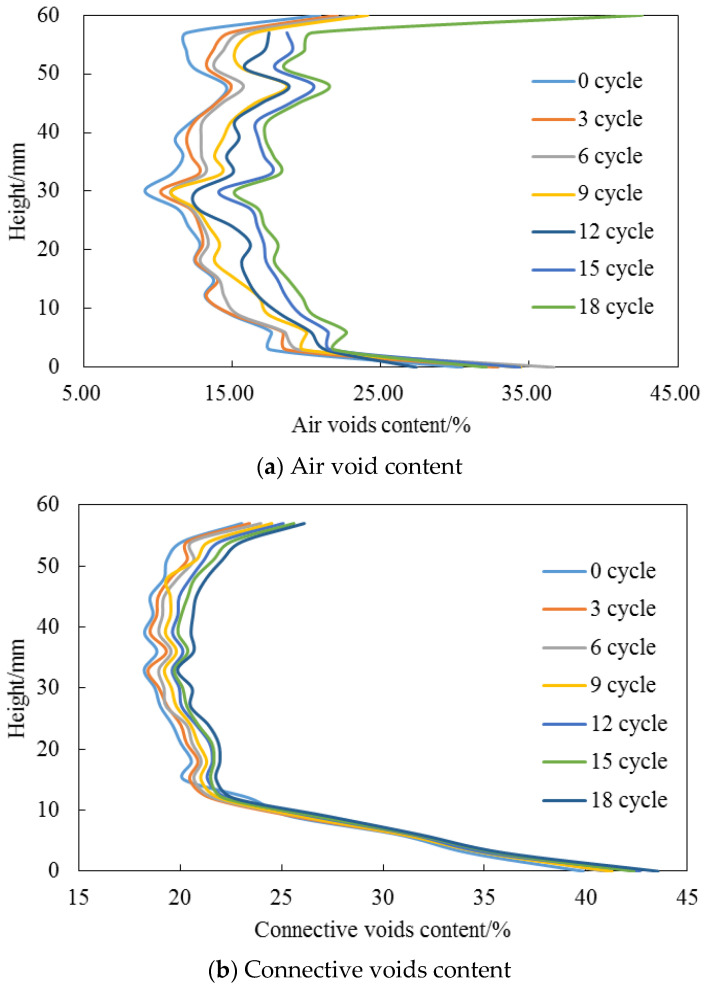

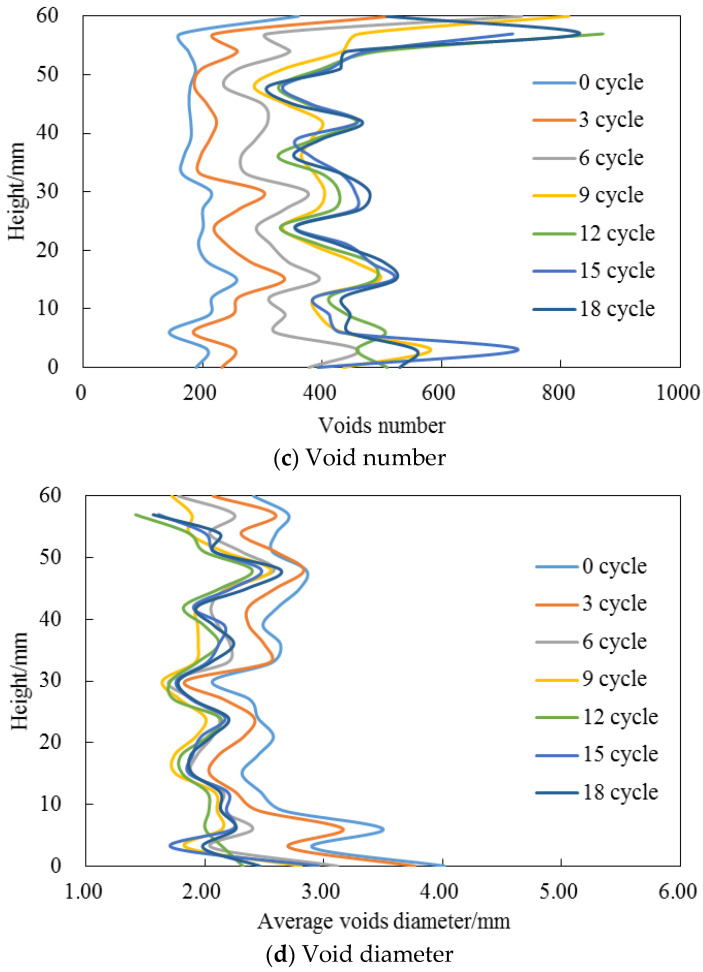
Pore structure degradation for OGFC mixtures under F-T cycles.

The variation of pore structure characteristics for AC and OGFC mixtures with various F-T cycles in T-1 test procedure were analyzed by utilizing X-ray CT images, as demonstrated in Figure 2 and Figure 3. A random sample of each asphalt mixture was taken for scanning. The variations in pore structure characteristics were analyzed from the curves by averaging the characteristics along the height direction with an interval of 3 mm. The average pore structure characteristics at the 10–50 mm depth are also listed in Table 5 and Table 6 to quantitatively demonstrate the effect of F-T cycles on the pore structure in asphalt mixtures. Note that our current experiments were relevant to [24,25], where the cracking mechanism can be investigated in future studies.

The curves shown in Figure 2 and Figure 3 clearly demonstrate similar trends for AC and OGFC mixtures under F-T action. Hence, in this section, the AC mixtures were taken as an example to explain the degradation of pore structure with F-T cycles. Figure 2a–c shows the degradation of (connective) void content and void number under F-T cycles for AC mixtures. A monotonically increase of the three parameters was observed with the F-T cycles increasing. Because the air voids are more uniform in the height of 10–50 mm, the mean of each parameter in this range is analyzed. Compared with that of 2.8% in the controlled case without F-T action, the average void content increased to 6.4% after 18 F-T cycles. Similarly, the percentage of the total void space had been increased to 64% possessed by the connective air void, suggesting the aggregation of voids. The F-T loading action also boosted the number of voids from 358 to 704, indicating the new voids formation under F-T action.

The degradation of void diameter under F-T cycles was shown in Figure 2d. At the first three F-T cycles, the void diameter decreased sharply. This phenomenon clearly indicated the dominant behavior of new voids formation. Then, from the third to ninth F-T cycles, the average void diameter increased stage by stage, suggesting the coalescing of existing voids with new formed voids under F-T loading action. After the ninth F-T cycle, the average void diameter was almost the same and not fluctuated with F-T action. This result showed the coexistence and mutually competition of new void formation and void merging with the increasing number of F-T cycles.

**Figure 4:**
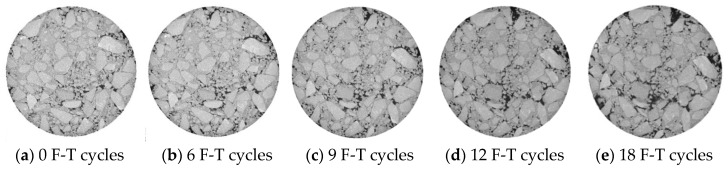
Comparison of image at a depth of 48 mm, before and after F-T test (AC mixtures).

**Figure 5:**
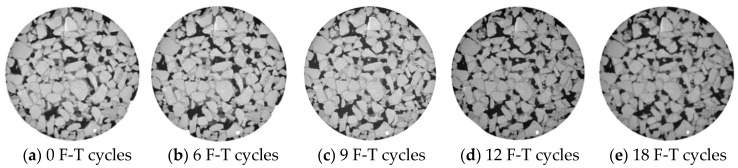
Comparison of image at a depth of 48 mm, before and after F-T test (OGFC mixtures).

The CT images of the pore structure at the depth 48 mm in sample is shown in Figure 4 and Figure 5. The degradation characteristics illustrated in these images fitted well with previously depicted findings listed above. For instance, new voids were formed in the middle part of sample under F-T action. The F-T cycles also made existing void dilate to form micro crack and aggravated the damage in asphalt mixtures.

#### 3.1.2. Information Entropy Evolution of Asphalt Mixtures under F-T Cycles

**Figure 6:**
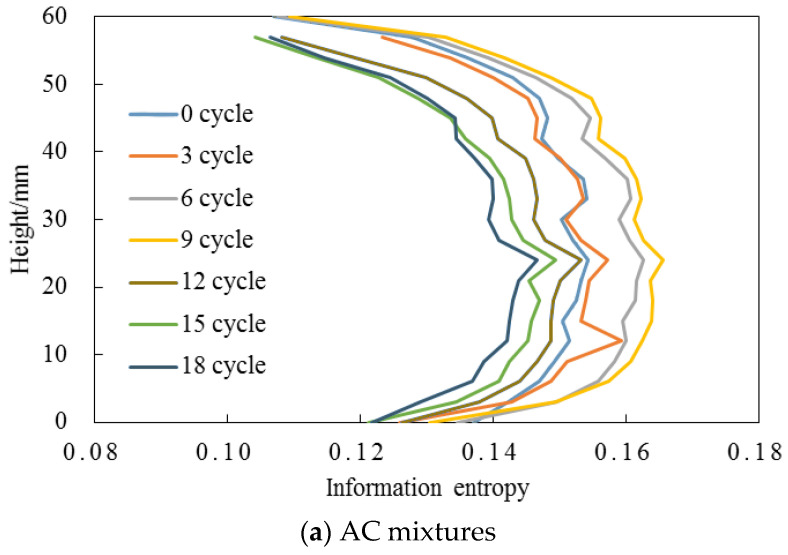

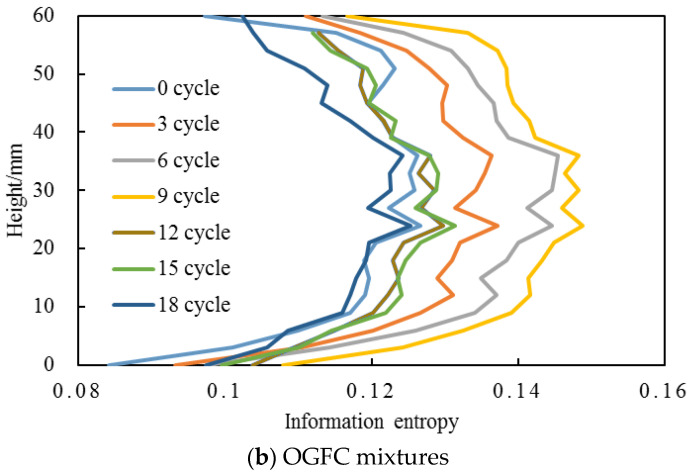
Changes of information entropy for asphalt mixtures under F-T cycles.

This section analyzes the information entropy in AC and OGFC mixtures during F-T actions and compared the results with the micro-scale pore structure evolution to examine the possibility in the F-T damage evaluation. Figure 6 presents the distribution of information entropy for AC and OGFC mixtures exposed to F-T cycles. The average information entropy for the samples at the 10–50 mm depth is listed in Table 7.

As illustrated in Figure 6, AC and OGFC mixtures shows increased information entropy with increasing F-T cycles at the initial phase of the experiment. A high information entropy always indicated a more severe debonding between asphalt and aggregate in asphalt mixtures as proposed by Zhang et al. [20,21,23]. Figure 6 shows that the information entropy of AC increased by 6.5% and OGFC increased by 17.4% with the F-T cycle increasing from 0 to 9. This phenomenon suggested that F-T action increased the degree of disorder of the aggregate-asphalt adhesion system and destroyed the adhesion between asphalt and aggregate which stripped the asphalt from the aggregate surface and resulted in the formation of new voids.

After the 9th F-T cycle, the information entropy significantly decreased. Compared with that of the 9 F-T cycle, the information entropy was reduced by 13.6% after the 18th F-T cycle. The sharp reduction of information entropy of the CT images meant the reduction of system disorder and the release of internal energy. Thus, this result indicated the existing voids coalescing with new voids and the formation of micro crack under F-T cycles. As a result of the formation of micro-crack, the energy accumulated in asphalt mixture by F-T cycles was released and the system disorder was consequently reduced.

Interestingly, the results derived from information entropy fitted well with the measured data shown in Figure 4 and Figure 5. New voids could be easily observed by comparing the CT image after the 6th F-T cycle with that after the 9th F-T cycle. And a significant void coalescing existed and some micro crack could be seen clearly from the image captured after the 12nd F-T cycle (Figure 4c and Figure 5c). In conclusion, the comparison of the results by information entropy analysis and pore structure analysis derived from CT images validated the accuracy and possibility of information entropy in the quantitative determination of F-T damage in asphalt mixtures. The information entropy was also used in the analysis in the next sections.

### 3.2. Effect of Test Conditions on the F-T Damage Propagation in Asphalt Mixtures

**Figure 7:**
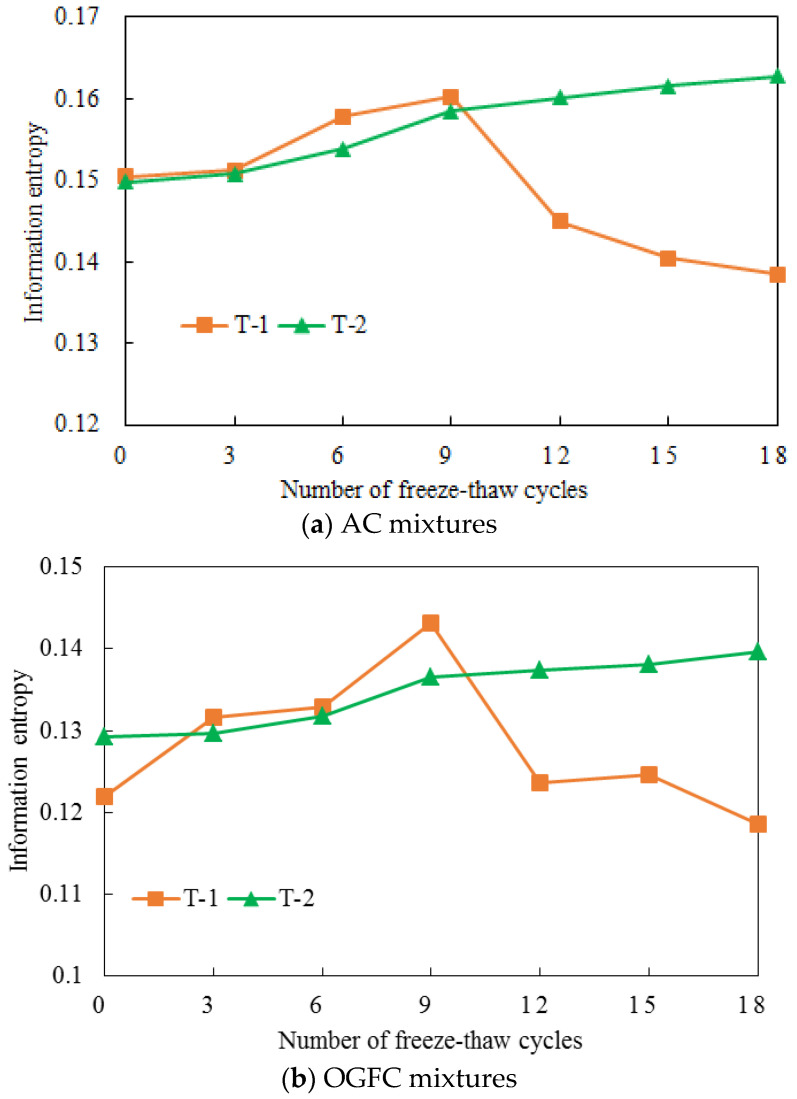
Comparison of information entropy of asphalt mixtures under various test conditions.

Test conditions are obviously one of the most important factors that affect the asphalt mixture F-T degradation [9,26,27]. Although most of previous studies were focused on the F-T analysis in asphalt mixtures with water, the non-negligible effect of vapor diffusion on the moisture induced damage in cold and drought regions gradually draw researchers’ attention [28,29,30]. Thus, a comparative discussion of two experimental conditions is presented in this part. As shown in Table 1, the specimens in the first test group were treated by water. The second test was set with a relative humidity of 90% with freezing in the air. Figure 7 shows the comparison of the information entropy derived from X-ray CT at the 10–50 mm depth with various experimental conditions and F-T cycles.

For the information entropy derived from X-ray CT (Figure 7) data, the curves for the AC and OGFC mixtures illustrated a similar trend under the two experimental conditions and F-T cycles. The curves of the specimens conditioned in a relative humidity of 90% increased monotonically with the F-T cycles. However, a parabola going downward existed in the curve of the samples treated by. This phenomenon indicated the differences of failure mechanism of asphalt mixtures between the two experimental conditions. Clearly, new void formation at the initial stage and void expansion and coalescing after the initial stage was the essential reason for the F-T failure of asphalt mixtures with the water condition, while new void formation at the asphalt-aggregate interface and not void coalescence played a predominant role on the F-T damage in the humid environment. Therefore, the F-T rebellion of asphalt pavements should be optimized in respect of its service environment.

## 4. Conclusions

To understand the fundamental thermodynamic behaviors of asphalt mixtures under F-T cycles, information entropy theory, an CT scanner and digital image processing technology were employed in the analysis. Changes in void structure of asphalt mixtures were captured by X-ray CT before and after F-T test. Based on the F-T experiments and the CT scanned images, the information entropy evolution of the asphalt mixtures under different F-T cycles was calculated and the relationship between the change of information entropy and the pore structure characteristics were discussed. The results illustrated the possibility of information entropy in the quantitative determination of F-T damage in asphalt mixtures.

Based on the limited F-T cycles, the information entropy evolution in two kinds of asphalt mixtures was analyzed. Interestingly, AC and OGFC mixtures showed increased information entropy with the increase in the number of F-T cycles during the initial stages of the test, suggesting that F-T action increased the degree of disorder of the aggregate-asphalt adhesion system and destroyed the adhesion between asphalt and aggregate. After the 9th F-T cycle, the information entropy significantly decreased, which indicated the existing voids coalescing with new voids and the formation of micro cracks during the F-T cycles.

Information entropy was also adopted to analyze the effect of test conditions on the F-T damage propagation in asphalt mixtures. A significant difference of failure mechanism existed in the asphalt mixtures conditioned by water and vapor moisture conditions. Results showed that new void formation at the initial stage and void expansion and coalescing after the initial stage was the essential reason for the F-T failure of asphalt mixtures with the water condition. New void formation at the asphalt-aggregate interface was the major factors that controlled the F-T damage in the humid environment. The next step of this study is to establish the equation of F-T damage of asphalt mixture under water and vapor moisture conditions based on information entropy. It is hoped that it can be used to quantitatively appraise the degree of F-T damage in asphalt mixtures and provide some reference.

## Figures and Tables

**Table 1:** The properties of the asphalt.

Index	Test Values	Requirement
Penetration 25 °C, 5 s, 100 g, (0.1 mm)	86.2	80~100
Softening point/(T/°C)	46.2	>45
Ductility/(cm)	>100	>100

**Table 2:** The properties of coarse aggregate.

Index	Sieve Size/mm				Requirement
	13.2	9.5	4.75	2.36	
Bulk specific gravity	2.892	2.752	2.627	2.730	≥2.5
Crushed stone value/%	15.3	15	14.9	15.0	≤28
Weared stone value/%	13.2	13.1	15.4	16.0	≤30
Adhesion level	5	5	5	5	≥4

**Table 3:** The properties of fine aggregate.

Index	Sieve Size/mm				
	1.18	0.6	0.3	0.15	0.075
Apparent specific gravity	2.789	2.772	2.766	2.778	2.752
Angularity/s	41				

**Table 4:** Detail of F-T test procedures.

	F-T Test Details			Mixture Type	Initial Air Void Content/%
Test Procedure	Step 1	Step 2	Step 3		
T-1	Water conditioned by vacuum saturation using a residual pressure of 98 kPa for 15 min	Freezing in the air at −18 °C for 16 h	Thawing in the water at 20 °C for 12 h	AC	3.5
				OGFC	21.1
T-2	Air conditioned by RH of 90% at 20 °C for 15 days before the first F-T test.		Thawing in the air with RH of 90 ± 1% at 20 °C for 12 h	AC	3.8
				OGFC	23.7

**Table 5:** Average pore structure characteristics at 10–50 mm depth (AC mixture).

Pore Structure Characteristics	Number of F-T Cycles						
	0	3	6	9	12	15	18
Air void content (%)	2.8	3.3	4.0	5.0	5.8	6.2	6.4
Void number	358	430	474	527	587	658	704
Average void diameter (mm)	0.95	0.79	0.91	0.97	0.99	0.97	0.95
Connective void content (%)	1.4	1.8	2.2	2.8	3.7	3.9	4.1

**Table 6:** Average pore structure characteristics at 10–50 mm depth (OGFC mixture).

Pore Structure Characteristics	Number of F-T cycles						
	0	3	6	9	12	15	18
Air void content (%)	21.0	21.5	21.8	22.7	23.7	24.2	24.4
Void number	196	238	309	382	405	418	425
Average void diameter (mm)	2.52	2.33	2.09	1.97	1.97	2.05	2.07
Connective void content (%)	20.6	20.8	21.0	21.4	21.7	21.9	22.2

**Table 7:** Changes of information entropy of asphalt mixtures under F-T cycles.

Mixture Type	Number of Freeze-Thaw Cycles						
	0	3	6	9	12	15	18
AC	0.1504	0.1511	0.1577	0.1602	0.1449	0.1404	0.1384
OGFC	0.1220	0.1316	0.1329	0.1432	0.1236	0.1246	0.1186
